# A review of the cues used for rejecting foreign eggs from the nest by the Eurasian blackbird (*Turdus merula*)

**DOI:** 10.1002/ece3.8886

**Published:** 2022-05-06

**Authors:** Andrew G. Fulmer, Mark E. Hauber

**Affiliations:** ^1^ 3054 Department of Psychology Fort Lewis College Durango Colorado USA; ^2^ Department of Evolution, Ecology, and Behavior School of Integrative Biology University of Illinois Urbana‐Champaign Urbana Illinois USA

**Keywords:** brood parasitism, common blackbird, common cuckoo, egg recognition, egg rejection

## Abstract

Avian brood parasitism is reproductively costly for hosts and selects for cognitive features enabling anti‐parasitic resistance at multiple stages of the host's breeding cycle. The true thrushes (genus *Turdus)* represent a nearly worldwide clade of potential hosts of brood parasitism by *Cuculus* cuckoos in Eurasia and Africa and *Molothrus* cowbirds in the Americas. The Eurasian blackbird (*Turdus merula*) builds an open‐cup nest and is common within much of the common cuckoo's (*C*. *canorus*) breeding range. While this thrush is known to be parasitized at most only at low rates by this cuckoo, the species is also a strong rejector of nonmimetic foreign eggs in the nest. Given their open‐cup nesting habits, we predict that Eurasian blackbirds primarily use visual cues in making a distinction between own and parasitically or experimentally inserted foreign eggs in the nest. We then provide a comprehensive and quantitative review of the literature on blackbird egg rejection studies. This review corroborates that vision is the primary sensory modality used by blackbirds in assessing eggs, but also brings attention to some other, less commonly studied cues which appear to influence rejection, including predator exposure, individual experience, stage of clutch completion, and maternal hormonal state. Blackbirds are also able to recognize and eject even highly mimetic eggs (including those of conspecifics) at a moderate rate, apparently relying on many of the same sensory cues. Although the cues involved in foreign egg recognition by Eurasian blackbirds do not appear specialized to nonmimetic cuckoo parasitism, we cannot differentiate between the possibility of egg rejection being selected by mostly conspecific parasitism or by the evolutionary ghost of a now‐extinct, mimetic cuckoo host‐race.

## Graphical abstract

We provide a comprehensive, quantitative review of the literature on cues used by Eurasian blackbirds (*Turdus merula*) to distinguish between own eggs and eggs experimentally or parasitically added to the nest. We find that visual cues are most commonly used, but also that indirect cues such as predator exposure, individual experience, and clutch completion stage are also used.

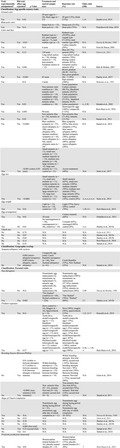

## 1 INTRODUCTION

Avian obligate brood parasites lay their eggs in the nests of other species. Parasitic nestlings consume resources otherwise aimed at the host parents’ own progeny in the nest, and in the case of the old world *Cuculus* cuckoos and several other lineages, these parasitic hatchlings evict or otherwise eliminate both unhatched host eggs and newly hatched host young from the nest (Kilner, 2005). As a result of these costs, there is strong selective pressure on hosts to evade or foil parasitism through defending their nests and/or the rejection of parasitic propagules in the nest (Soler, 2017).

Parasitism can be dampened at each stage of nesting; nests can be constructed in such a way as to deter parasitic laying or prevent ejection of host chicks by parasitic chicks (e.g., Grim et al., 2011), breeding can be initiated at different times of year than when potential parasites breed (e.g., Anderson et al., 2013), brood parasite adults can be recognized and driven off or their presence can cause the abandonment of a nest (e.g., Briskie et al., 1992; Lyon & Gilbert, 2013), eggs laid can be ejected or their presence can cause the abandonment of the nest (Antonov et al., 2007), as can hatched parasitic chicks (e.g., Grim, 2011) which may also fail to thrive under host parental care (e.g., Grim et al., 2011). We focus here on the effective anti‐parasitic behavior at the egg rejection stage in one of the most commonly experimentally studied potential host species, the Eurasian Blackbird (*Turdus merula*).

Hosts risk fitness losses by incorrectly rejecting or damaging their own eggs during attempts to eject foreign eggs and can be expected to evolve a perceptive suite appropriate for discriminating between own and parasite eggs and retaining their own eggs (e.g., Turner & Hauber, 2021). Properties of the focal egg (e.g., color, maculation, size, shape), conditions of nest (disruption, re‐arrangement of eggs, predator or parasite presence), and conditions of the host bird (parental stress, prior experience of own or parasitic egg appearance, hormonal state, stage in the breeding season, laying process, or final clutch size) may all influence likelihood of rejection (Turner & Hauber, 2021). Additionally, hosts may use perceptual differences in the properties of a parasitically laid egg relative to own eggs as a rejection cue (Davies & Brooke, 1989; Rothstein, 1975).

Thrushes in the genus *Turdus* are native to five continents and have been established as successful introduced populations on a sixth (Cassey et al., 2008; Voelker et al., 2007). Eurasian thrushes are rarely parasitized by the often sympatric common cuckoo (*Cuculus canorus*), which more regularly parasitizes smaller passerines, such as reed warblers (*Acrocephalus* spp.), meadow pipits (*Anthus pratensis*), pied wagtails (*Motacilla alba*), and common redstarts (*Phoenicurus phoenicurus*) (Davies & Brooke, 1989; Grim et al., 2011). Many of the cuckoo gentes that parasitize these hosts have evolved egg coloration mimetic to their respective host species, but no gens is known to currently exist which mimics turdid egg colors (Grim et al., 2011). Despite this, many European thrushes reject experimentally introduced foreign eggs from their nests, including in their native (e.g., Grim et al., 2011; Moskát et al., 2003) and introduced ranges (e.g., Hauber et al., 2014). In turn, American robins (*T*. *migratorius*) are robust rejectors of eggs parasitically laid by the sympatric and obligate brood parasitic brown‐headed cowbird (*Molothrus ater*) (e.g., Croston & Hauber, 2015). In East Asia, there is a wider range of brood parasites and local thrushes may be more vulnerable to parasitism (Yi et al., 2020). The gray‐backed thrush (*T*. *hortulorum*) is a strong rejector of immaculate blue model eggs (Yang et al., 2019), while experimentally parasitized chestnut thrushes (*T*. *rubrocanus*) have exhibited more moderate egg rejection behavior (Yi et al., 2020). Brood parasitism has not been extensively examined in neotropical *Turdus* (Davanço et al., 2012) but Astié & Reboreda (2005) found that the South American creamy‐bellied Thrush (*T*. *amaurochalinus*) was a relatively weak rejector of model shiny cowbird (*M*. *bonariensis*) eggs, while the rufous‐bellied thrush (*T*. *rufiventris*) is a relatively strong rejector of the same (Lichtenstein, 2001; Sackmann & Reboreda, 2003). The African kurrichane thrush *T*. *libonyana* and the southern olive thrush *T*. *olivaceous* are both strong rejectors of nonmimetic eggs, while only the kurrichane thrush also rejects mimetic eggs (at the rate of 60%) (Honza et al., 2005).

Whether this resistance to parasitism, where it appears in the thrushes, is the result of a history of interspecific brood parasitism or conspecific is the subject of debate (Ruiz‐Raya et al., 2016; Samaš et al., 2014a, 2014b; Soler, 2014; Yi et al., 2020). This is because the Eurasian blackbird (hereafter: blackbird) and the song thrush (*T*. *philomelos*) exhibit high levels of conspecific egg rejection relative to other studied passerines, suggesting that adaptations preventing parasitism may have evolved at least in part as a response to intra/conspecific parasitism (Samaš et al., 2014a). The same suggestion has been made of the redwing (*T*. *iliacus*) (Grendstad et al., 1999 *in* Grim & Honza, 2001). However, the suitability of a host species for obligate brood parasitism, formerly recorded dichotomously (Davies & Brooke, 1989) may be better understood as occurring along a spectrum (Stokke et al., 2018). Finally, regarding the evolutionary history of egg rejection in the genus *Turdus*, the blackbird's status within the Eurasian turdid radiation has been somewhat unclear; while the species has typically been considered a basal member of the clade, that position was not strongly supported by a Bayesian analysis (Voelker et al., 2007) and more recent multilocus work puts it in a western palearctic clade as a sister taxon to *T*. *iliacus* (Batista et al., 2020).

Although interspecific brood parasitism rates for the blackbird (along with the song thrush) appear to be higher than for other Eurasian thrush species (Samaš et al., 2014a), blackbird nests are also rarely parasitized by common cuckoo relative to other sympatric, similarly abundant, open‐cup nesting passerine species (e.g., Davies & Brooke, 1989). Blackbird nests are easily accessible for parasitism (Davies & Brooke, 1989), but cuckoo chicks in these nests exhibit signs of malnutrition, suggesting that the diet fed by blackbirds to young is not suitable for cuckoos (Grim, 2006; Grim et al., 2011). In the closely related song thrush, which also uses an open‐cup nest, the mud‐built nest‐wall structure appears to impede eviction of host young (Samaš et al., 2014a), whereas cuckoo offspring in blackbird nests have been observed to successfully evict host young (Grim et al., 2011). When blackbird host parents have not deserted (as they did in Grim et al., 2011) the cuckoo chicks still fail to thrive and may suffer fatal malnutrition (Grim, 2006). Interactions between host and parasite young in the nest appear to be an important limiting factor in suitability, and it has been suggested that the traditional approach of studying particular host traits in isolation does not account for the diversity or potential interactivity of host anti‐parasite discrimination tactics (Grim et al., 2011).

For open‐cup nesting host species, visual cues are the most likely mechanism of egg rejection (e.g., Samaš et al., 2021; Turner & Hauber, 2021). Given that there is no extant cuckoo gens with eggs mimetic of the color/pattern of true thrush eggs, conspicuous visual cues such as eggshell color, maculation, shape, and size could potentially be sufficient for Eurasian turdid species to recognize nonmimetic parasitic eggs over the need of other, non‐visual sensory modalities. The differences between many cuckoo gentes' and the blackbird's eggs in both coloration and size are considerable (e.g., Moskát et al., 2003; Soler et al., 2015) and would be highly noticeable to the blackbird visual system (e.g., Hanley et al., 2017). If selective pressure from intra/conspecific brood parasitism shaped the blackbird egg recognition/rejection suite of cognitive traits and behaviors, we would expect that heterospecific eggs would be even more easily recognized than conspecific eggs, since a finer level of detail must be processed to distinguish conspecific eggs from own than heterospecific eggs from own. Because heterospecific eggs differ along more dimensions than conspecific eggs, we expect that a correspondingly wider variety of cues, either visual or multimodal, would “give away” heterospecific eggs (e.g., Ruiz‐Raya et al., 2016; Samaš et al., 2014a).

## 2 METHODS

Following the effect‐sized‐based quantitative methodology used by Turner & Hauber (2021) in reviewing the literature on American robin rejection of parasitic/experimentally introduced eggs, we surveyed published work recording the egg rejection decisions of Eurasian blackbirds in response experimentally parasitized clutches. Papers included in these data were gathered from Google Scholar using the search terms “European Blackbird,” “Eurasian Blackbird,” “*Turdus merula*,” “egg rejection,” “egg ejection,” “egg recognition,” and “brood parasitism.” All papers accessed (*N* = 21) featured the assessment of experimental egg rejection by Eurasian blackbirds regarding natural or specifically manipulated egg trait/cues. We included work from 1989 to 2021. These data, including our calculations of the effect sizes (odds ratios), are summarized in Table 1, indicating the authors, focal egg trait/cue, presence/absence of experimental manipulation of egg traits/cues, whether or not the trait affected egg rejection, the treatment and control sample sizes, the rejection rate per treatment, and the odds ratio (OR), that was calculated using the formula:

OR=AD/BC

A: Number of eggs rejected in the experimental treatment, B: number of eggs rejected in the control treatment, C: number of eggs accepted in the experimental treatment, and D: number of eggs accepted in the control treatment.

**TABLE 1:** Summary of the accessed published studies on the egg rejection behavior of the Eurasian blackbird

Trait experimentally manipulated?	Did trait affect egg rejection?	*p* Value	Treatment and control sample sizes	Rejection rate (%)	Odds ratio (OR)	Source
Classification: Egg‐specific sensory traits						
Ground color						
Yes	Yes	.02	20‐spot eggs (*n* = 30), black eggs (*n* = 27)	20 spot (15%), black (17%)	1.16	Hauber et al. (2014)
Blunt pole color						
Yes	Yes	N/A	Sharp Pole (*n* = 15), blunt pole (*n* = 16)	Sharp pole (33.3%), blunt pole (75%)	N/A	Polačiková and Grim (2010)
Color and Size						
Yes	Yes	<.01	Redstart type (*n* = 22), pied wagtail type (*n* = 7), reed warbler type (*n* = 5)	Redstart type (59.09%), pied wagtail type (71.43%), reed warbler type (60%)	N/A	Davies and Brooke (1989)
Yes	‐	N/A	6 nests	Cuckoo mimetic (50%)	N/A	Grim and Honza (2001)
Yes	No	.12	130 nests	Blue (65.7%), spotted (50%)	N/A	Grim et al. (2011)
Yes	Yes	.001	Long‐tailed cuckoo (*n* = 18), shining cuckoo (*n* = 13), host‐mimetic European cuckoo (*n* = 12)	Long‐tailed Cuckoo mimetic (78%), shining cuckoo mimetic (85%), European cuckoo mimetic (8%)	N/A	Hale and V. Briskie (2007)
Yes	Yes	<.001	82 nests	Brown gradient (86.98 + 0.61%), blue green gradient (66 + 3.18%)	N/A	Hanley et al. (2017)
Yes	‐	N/A	2 nests	Pipit mimetic (100%)	N/A	Moksnes et al. (1991)
Yes	Yes	0.03	Nonmimetic rural condition (*n* = 26), mimetic rural condition (*n* = 14), nonmimetic urban condition (*n* = 43), mimetic urban condition (*n* = 36)	Forest population: Cuckoo mimetic (88.5%), Blackbird mimetic (28.6%). Urban population: Cuckoo mimetic (53.5%), blackbird mimetic 44.4% (urban population)	∞, 2.56	Moskát et al. (2003)
Yes	Yes	.001	90 nests	Heterospecific eggs (100%), conspecific (<13%)	N/A	Ruiz‐Raya et al. (2016)
Yes	Yes	.03	Nonmimetic blue (*n* = 56), mimetic (*n* = 16)	Nonmimetic blue (71.4%), mimetic (12.5%)	N/A	Samaš et al. (2011)
Yes	Yes	<.0001	Blue condition: sympatric (*n* = 37), micro‐allopatric (*n* = 280), macro‐allopatric (85); Spotted condition: sympatric (*n* = 16), micro‐allopatric (*n* = 64), macro‐allopatric nests (*n* = 24)	Blue sympatric (50%), blue micro‐allopatric (75%), blue macro‐allopatric (60%). Spotted sympatric (30%), spotted micro‐allopatric (40%), spotted macro‐allopatric (20%)	N/A	Samaš et al. (2014)
Yes	Yes	<.001 (color); .03 (size)	Small mimetic (*n* = 11), small nonmimetic (*n* = 11), medium mimetic (*n* = 14), medium nonmimetic (*n* = 14), large mimetic (*n* = 12), large nonmimetic (*n* = 11), control (*n* = 21)	Across treatments (48%)	N/A	Soler et al. (2017)
Egg size						
Yes	Yes	<.0001	Small mimetic (*n* = 11), small non‐mimetic (*n* = 11), medium mimetic (*n* = 14), medium nonmimetic (*n* = 14), large mimetic (*n* = 12), large nonmimetic (*n* = 11), control (*n* = 21)	Small mimetic (65%), small non‐mimetic (100%), medium mimetic (30%), medium nonmimetic (70%), large mimetic (0%), large nonmimetic (10%), control (0%)	∞,∞,∞,∞,∞,∞	Soler et al. (2015)
Egg weight						
Yes	Yes	.01	Light (*n* = 13), heavy (*n* = 14), control (*n* = 13)	Light (75%), heavy (20%), control (70%)	1.29, 0.1	Ruiz‐Raya et al. (2015)
*Egg arrangement*						
Yes	Yes	.01	20 nests	Cuckoo mimetic (60%)	N/A	Polačiková et al. (2013)
Yes	No	.01	No rearrangement (*n* = 30), rearrangement (*n* = 30), control (*n* = 19)	Constant (65%), rearranged (80%), control (0%)	∞,∞	Hanley, Samaš, Hauber, et al. (2015)
Clutch size						
No	No	.74	N/A	N/A	N/A	Grim et al. (2011)
No	No	.97	N/A	N/A	N/A	Hanley, Samaš, Hauber, et al. (2015)
No	No	.69	N/A	N/A	N/A	Hanley, Samaš, Heryán, et al. (2015)
No	No	.15	N/A	N/A	N/A	Ruiz‐Raya et al. (2016)
No	No	.53	N/A	N/A	N/A	Samaš et al. (2014)
Classification: Geographic overlap						
Sympatry/allopatry with cuckoos						
No	Yes	.002 (micro‐allopatry/sympatry); .005 (micro‐allopatry, macro‐allopatry)	Conspecific egg (real): Czech Republic (sympatry) (*n* = 66), New Zealand (allopatry) (*n* = 41)	Czech Republic (15%), New Zealand (35%)	N/A	Samaš et al. (2014)
Classification: Parental traits						
Nest disruption						
Yes	No	N/A	Nonmimetic egg replacement (2), nonmimetic no replacement (7), mimetic egg replacement (6), mimetic no replacement (10)	Nonmimetic egg replacement (100%), nonmimetic no replacement (57.14%), mimetic egg replacement (33%), mimetic no replacement (20%)	5.49	Davies and Brooke (1989)
Yes	Yes	.002	"Not flushed" condition (*n* = 69), flushed condition (*n* = 224)	"Not flushed" (70%), "flushed" (80%)	2.7	Hanley, Samaš, Heryán, et al. (2015)
Predator exposure						
Yes	Yes	N/A	Dove control (*n* = 16), Magpie (*n* = 16), Sparrowhawk (*n* = 13)	Dove (50%), magpie (56%), sparrowhawk (15%)	1.21, 0.17	Roncalli et al. (2019)
Yes	No	.57	Turtle dove model/conspecific egg (*n* = ~17), blackbird model/conspecific egg (*n* = ~17), cuckoo model/conspecific egg (*n* = ~17), dove model/heterospecific egg (*n* = ~17), blackbird model heterospecific egg (*n* = ~17), cuckoo model/heterospecific egg (*n* = ~17)	Turtle dove model/conspecific egg (~15%), blackbird model/conspecific egg (~5%), cuckoo model/conspecific egg (~15%), dove model/heterospecific egg (~85%), blackbird model heterospecific egg (~90%), cuckoo model/heterospecific egg (100%)	0.30, 1, 1.59, ∞	Ruiz‐Raya et al. (2016)
Breeding Season (Between/Within)						
No	Yes	.01 (within vs between attempts); .15 (within vs between seasons); .48 (between attempts vs between seasons)	Within breeding attempts (*n* = 73), between breeding attempts (*n* = 23), between breeding seasons (*n* = 19)	Within breeding attempts: first trial (80%), second trial (~82%). Between breeding attempts: first trial (~78%) second trial (70%). Between breeding seasons: first trial (~73%), second trial (90%)	N/A	Grim et al. (2014)
No	Yes	<.0001 (nonmimetic); .03 (mimetic)	Nonmimetic blue (*N* = 41), mimetic first trial (*N* = 6), mimetic second trial (*N* = 8)	Nonmimetic blue: first trial (83%), second trial (78%), mimetic: first trial (14%), second trial (25%)	N/A	Samaš et al. (2011)
*Stage of Clutch Completion*						
Yes	No	N/A	Nonmimetic egg during laying period (*n* = 21), nonmimetic egg after day of clutch completion (*n* = 11)	Nonmimetic egg during laying period (47.62%), Nonmimetic egg after day of clutch completion (81.82%)	N/A	Davies and Brooke (1989)
No	No	.23	N/A	N/A	N/A	Grim et al. (2011)
No	No	.29	N/A	N/A	N/A	Hanley, Samaš, Hauber, et al. (2015)
No	No	.14	N/A	N/A	N/A	Hanley, Samaš, Heryán, et al. (2015)
No	Yes	.009 (first trial) .52 (second trial)	N/A	N/A	N/A	Samaš et al. (2011)
No	Yes	.4	N/A	N/A	N/A	Samaš et al. (2014)
Prolactin production (maternal)						
Yes	Yes	.01	Bromocriptine treated females (*N* = 16), placebo treated females (*N* = 15)	Bromocriptine treated females (75%), placebo treated females (*N* = 30%)	7	Ruiz‐Raya et al. (2021)

Nest desertion cannot be equated with egg rejection in blackbirds (Soler et al., 2015), and although a number of studies include this information (e.g., Hanley, Samaš, Heryán, et al., 2015; Hauber et al., 2014; Soler et al., 2015), we did not consider it in this review as a response to experimental brood parasitism.

## 3 RESULTS

### 3.1 Effect sizes

Odds ratios were calculated where sufficient information (sample sizes for experimental and control groups) in papers was available. We provide these as a mechanism for future studies to compare or meta‐analyze the results of the papers reviewed.

#### 3.1.1 Egg traits

##### Ground color

In a recent and large‐scale meta‐analysis conducted across all hosts and brood parasites (Samaš et al., 2021), eggshell color was the most frequently investigated trait in obligate brood parasitism experiments. Since model cuckoo eggs painted to be mimetic or nonmimetic to blackbird eggs were frequently used in experimenting with Eurasian blackbird populations, and blackbird eggs are larger than cuckoo eggs (Moskát et al., 2003), many of the tests in blackbirds tested both color and size (see below, 3.1.1.3). Eurasian blackbird eggs are blue‐green with varying degrees of red‐brown maculation (Cassey et al., 2008; Samaš et al., 2011). Hauber et al. (2014) manipulated blackbirds' own eggs and found that overall rejection (exclusive of nest desertion) was not statistically different (~2%) for all‐black than for artificially spotted eggs. All other experiments involving egg color also manipulated model egg size (3.1.1.3).

##### Blunt pole color

Color discrimination may be more heavily weighted to the egg's blunt pole (Polačiková et al., 2007). Evidence for greater salience of the cues in this region appears in a variety of species (Polačiková & Grim, 2010), including the song thrush (Polačiková & Grim, 2010) (though not the American robin: see Hauber et al., 2021). As with other thrushes, maculation is denser at the blunt pole of blackbird eggs, and painting this pole immaculate blue led to significantly higher rejection rates than applying the same paint to the sharp pole (Polačiková & Grim, 2010).

##### Color and size

Manipulations featuring extant cuckoo‐mimetic eggs (in particular, models painted to resemble the eggs of the gens parasitizing the redstart) were the most common experimental paradigm (Davies & Brooke, 1989; Grim & Honza, 2001; Hanley et al., 2017; Hanley, Samaš, Hauber, et al., 2015; Hanley, Samaš, Heryán, et al., 2015; Samaš et al., 2011, 2014). These differ from blackbird eggs in both color and size, being immaculate blue and substantially smaller than blackbird eggs (Moskát et al., 2003). As expected, such cuckoo‐mimetic models were rejected at higher rates than are blackbird mimetic cuckoo‐sized models (e.g., Davies & Brooke, 1989; Grim & Honza, 2001; Moksnes et al., 1991; Moskát et al., 2003; Samaš et al., 2011, 2014a).

Model cuckoo eggs painted to be along the brown end of an egg color gradient also elicited higher rejection responses than eggs toward the blue end of the gradient (Hanley et al., 2017). In these experiments, only one size of model egg was presented. In Hale and Briskie (2007), introduced blackbirds in New Zealand, without sympatric common cuckoos, rejected nonmimetic model eggs of two native species of cuckoo at higher rates than they did these cuckoo‐sized egg models painted in blackbird egg colors. As these native cuckoo model eggs were themselves of different sizes from one another, these authors concluded that the higher rate of rejection was based on visible appearance more than on size.

Soler et al. (2015) manipulated both color and size, painting eggs of three different sizes either nonmimetic red or mimetic to a blackbird's egg. Experimentally introduced egg sizes included a set larger than blackbird eggs (common quail *Coturnix coturnix* eggs), a set of blackbird‐sized eggs, and a set smaller than blackbird eggs (house sparrow *Passer domesticus* eggs). Small nonmimetic eggs were ejected at a higher rate than any other egg type, and medium nonmimetic eggs were ejected more frequently than medium mimetic eggs. Large nonmimetic eggs were rejected at a low rate (and large mimetic eggs were never ejected). The authors suggest that this is in part informed by the difficulty blackbirds, as grasp‐ejectors, would have in grasping the large eggs with their beaks; desertion, instead, appears to be associated with large foreign eggs, as these eggs are deserted (marginally) significantly more often than controls (small eggs were never deserted and large eggs were deserted17.4% of the time) (Soler et al., 2015). This is similar to the behavior exhibited in the Bonelli's warbler (*Phylloscopus bonelli*) (Roncalli et al., 2017).

##### Egg size

Blackbird eggs are substantially larger than cuckoo gentes' eggs (Moskát et al., 2003), but within the capacity of the blackbirds to grasp and eject them both (Soler et al., 2017). In Soler et al. (2015), large blackbird color‐mimetic (painted common quail) eggs were never ejected, and small blackbird color‐mimetic (painted house sparrow) eggs were ejected more often than blackbird eggs, suggesting that a size‐based rejection criterion exists within the set of physically removable eggs for blackbirds (see 3.1.1.3).

##### Egg weight

Blackbird eggs are about twice as heavy as cuckoo eggs (Moskát et al., 2003). Ruiz‐Raya et al. (2015) painted model eggs a nonmimetic red and filled them with a sand‐silicone mixture to alter weights. Heavier eggs were, on average, 49% heavier than natural blackbird eggs and lighter eggs were, on average, 55% lighter than natural blackbird eggs. Heavier model eggs were rejected less often than either normal (average blackbird egg weight) or light weight model eggs.

##### Egg arrangement

Blackbirds with relatively consistent blunt pole distances among their eggs exhibited higher rates of rejection than did those with more variable arrangement, leading to the suggestion of an “egg‐arrangement hypothesis” (Polačiková et al., 2013). However, the experimental manipulation of egg arrangement in a study assessing this hypothesis showed no significant difference between undisturbed and rearranged nests in rejection rate (Hanley, Samaš, Hauber, et al., 2015).

##### Clutch size

Clutch size at completion was investigated in several studies (Grim et al., 2011; Hanley, Samaš, Hauber, et al., 2015; Hanley, Samaš, Heryán, et al., 2015; Ruiz‐Raya et al., 2016; Samaš et al., 2014). Grim et al. (2011) even considered this measure a proxy for host quality. However, clutch size was not found to influence rejection in any of these cases.

#### 3.1.2 Geographic overlap

##### Sympatry/allopatry with cuckoos

Sympatry with cuckoos should create selective pressure for resisting interspecific brood parasitism. Samaš et al. (2014) compared the responses of blackbirds in Czech Republic populations sympatric (Czech Republic rural area with cuckoos) or micro‐allopatric (Czech Republic urban area without cuckoos) with cuckoos and New Zealand populations, which are macro‐allopatric with common cuckoos (no common cuckoos are in New Zealand and no native cuckoo parasitism of blackbirds occurs there, either). Contrary to predictions, in this study, there was no difference in rejection of model eggs between macro‐allopatric and sympatric blackbird populations, and the micro‐allopatric population exhibited higher rejection rates than sympatric or macro‐allopatric populations.

#### 3.1.3 Parental traits

Eurasian blackbirds exhibit biparental care: both sexes perform nest visits and provision chicks, though females provide nearly all incubation (Chamberlain et al., 1999; Magrath, 1988; Préault et al., 2005). Surprisingly, males have also been observed ejecting parasitic eggs, albeit at far less frequently than females (2.3% vs 97.7%) (Ruiz‐Raya et al., 2019).

##### Nest disruption

Nest disruption by experimenters includes flushing incubating blackbirds from nests when model eggs are added to replace or augment host eggs in the nest during parasitism. Flushing was found to influence rejection rates, with flushed blackbirds more likely to reject nonmimetic model eggs (Hanley, Samaš, Heryán, et al., 2015).

##### Predator exposure

Predator threat specific to adults and predator threat specific to nests can be expected to have different implications for the attention paid to individual eggs (Roncalli et al., 2019). Parents under direct threat would, in this model, reduce their egg rejection rates due to increased focus on anti‐predator behavior, while parents presented with threats to the nest should increase attention to eggs and thereby increase rejection of parasitic eggs. Roncalli et al. (2019) tested this hypothesis with mimetic model eggs and multimodal playback (a predator model and accompanying audio recording) and found that egg rejection decreased with adult specific predator threat (a model sparrowhawk *Accipiter nisus*) but was not affected by nest specific threat (a model magpie *Pica pica*). The effect of an adult‐specific predator threat decreased as the breeding season reached its height. Ruiz‐Raya et al. (2016) experimentally parasitized blackbird nests with either heterospecific or conspecific egg models (3.1.1.2) and exposed the parents to dummies representing a potential nest predator/parasite (a common cuckoo), a dummy blackbird, or a dummy neutral sympatric, a turtle dove (*Streptopelia turtur*). Rejection was much more frequent in response to heterospecific eggs under all conditions, with the dummy cuckoo eliciting highest rejection rate, followed by the blackbird, followed by the turtledove (these differences were not significant, however). This trend (there was not a significant effect of dummy type for either type of egg model) was not the same with conspecific eggs, where exposure to the cuckoo and turtledove elicited the same level of rejection behavior; the blackbird model elicited the lowest level (Ruiz‐Raya et al., 2016).

##### Breeding season (between/within)

Blackbirds are seasonally socially monogamous, site‐faithful birds in which multiple breeding attempts in a season are a possibility for females in good condition (Faivre et al., 2001). These traits permit individual plasticity or repeatability in egg decisions both within and between breeding seasons. Grim et al. (2014) tested within breeding attempt, between breeding attempt, and between breeding season repeatability with immaculate blue eggs. Within breeding attempts, there was high repeatability, with only two of 73 switching rejection behaviors. There was no effect of whether eggs were introduced during laying or incubation. Between breeding attempts, within a breeding season, only three of 23 altered rejection behavior. Between breeding season repeatability was not significantly different from within‐breeding season repeatability, when adjusted for covariates. Between breeding seasons, three of 19 females changed their response type (Grim et al., 2014). Samaš et al. (2011) also examined the effect of experience from prior clutches within a breeding season (two consecutive broods) and found high individual consistency. In the nonmimetic egg condition, 39 of 41 females exhibited consistent ejection behavior. In the mimetic egg condition, females of the eight nests for which there were data showed no switching in rejection responses (Samaš et al., 2011).

##### Stage of clutch completion

Blackbirds typically lay three eggs during each laying period (Roncalli et al., 2019), although individuals in some populations lay four eggs, and this may be a useful proxy for host fitness (Grim et al., 2011). An increase in the number of eggs following clutch completion should be a significant cue to nest parasitism. Consistent with this, nonmimetic blue eggs added to nests during the laying period were rejected at a rate of 47.62%, while nonmimetic blue eggs added the day following clutch completion were rejected at a rate of 81.82% (Davies & Brooke, 1989). Samaš et al. (2011) found rejection to be higher at later in the brooding process, and Samaš et al. (2014) found that rejection of nonmimetic eggs occurred was also more likely in more advanced nesting stages.

##### Prolactin production (maternal)

Prolactin is a hormone depressed in expression by stress and having a strong regulatory influence on avian parental care, including incubation. It is elevated at least during the beginning, and sometimes throughout, the egg laying period. Higher levels are sustained for a longer portion of the parental care period of birds with altricial young than in those with precocial young (e.g., Angelier & Chastel, 2009). Caretakers (including alloparents in cooperatively breeding scrub jays *Aphelocoma coerulescens*) have higher levels of prolactin than non‐care‐provisioning parents/members of the breeding group (Schoeh et al. 1996 *in* Angelier & Chastel, 2009). Experimentally lowered prolactin levels reduce other forms of alloparenting (such as kidnapping in Emperor penguins *Aptenodytes forsteri*) and lowered prolactin is also associated with egg abandonment (e.g., Angelier & Chastel, 2009). Subcutaneous time‐release dopamine receptor antagonist pellet implanted just after clutch completion were used by Ruiz‐Raya et al. (2021) to lower the circulating prolactin levels of female blackbirds. Individuals with lowered prolactin were experimentally parasitized with mimetically painted, natural blackbird eggs, and rejected these eggs at a higher rate than placebo implanted individuals (Ruiz‐Raya et al., 2021).

## 4 DISCUSSION

As expected, blackbirds rely heavily on visual cues to make egg rejection choices. Ten out of the 21 articles included in our review used immaculate blue eggs, similar to those laid by the cuckoo gens parasitizing redstarts (Davies & Brooke, 1989; Grim & Honza, 2001; Grim et al.; 2014; Grim et al., 2011; Hanley, Samaš, Hauber, et al., 2015; Hanley, Samaš, Heryán, et al., 2015; Moksnes et al., 1991; Polačiková et al., 2013; Samaš et al., 2011, 2014a). This is of course due to the pattern that experimental studies have also most frequently focused on visual eggshell cues (Davies & Brooke, 1989; Grim & Honza, 2001; Hale & V. Briskie, 2007; Hauber et al., 2014; Moskát et al., 2003; Polačiková & Grim, 2010; Ruiz‐Raya et al., 2016; Samaš et al., 2011, 2014a), though size assessment could also involve tactile cues (Turner & Hauber, 2021), and it is suggested that the extremely low rejection rates of larger‐than‐natural eggs may be influenced by the physical difficulty of ejecting such eggs from a deep cup nest (Soler et al., 2015). Blackbirds exhibit moderate intraclutch variation in egg size, with last eggs being somewhat larger than the clutch average (Slagsvold et al., 1984). In a review of a wide range of avian clades, Krist (2011) found that egg size was likely positively correlated with offspring survival, and so blackbirds may have further incentive to retain large eggs in their natural clutches (Soler et al., 2017). Egg arrangement would also likely be judged visually rather than in a tactile modality (Polačiková et al., 2013), though this was not consistently found to influence rejection in an experiment (Hanley, Samaš, Hauber, et al., 2015). Polačiková et al. (2013) suggest that rearranged eggs may be a cue for hosts to engage in more targeted egg recognition behaviors and that the new spatial arrangement may in this way indirectly lead to rejection.

Specific experiences of focal individuals have also been identified as influencing rejection behavior; flushing from nests (Hanley, Samaš, Heryán, et al., 2015) increased rejection, as did exposure to a predator (Roncalli et al., 2019). The role of prior experience with parasitism within and between breeding seasons was marginally nonsignificant in Grim et al. (2014) (see 3.1.3.4). This is not surprising, since blackbirds exhibit strong inter‐individual variation and high intra‐individual repeatability in egg rejection decisions (Polačiková et al., 2013). Rejection rates were substantially higher immediately after the last host egg is laid than during the laying period, in the one study that included the influence of the stage of clutch completion (Davies & Brooke, 1989). Maternal hormonal state also plays a role, with prolactin suppression increasing the rate of egg rejection (Ruiz‐Raya et al., 2021).

Blackbirds appear to be moderately robust rejectors of diverse foreign egg types, including conspecific eggs (e.gDavies & Brooke, 1989; Moskát et al., 2003; Samaš et al., 2011), but these results suggest that they are not particularly specialized to reject cuckoo parasitism; this is not surprising as there is no cuckoo extant gens specialized to blackbird parasitism to drive a coevolutionary relationship. Cuckoo hatchlings do not typically survive to fledging when reared by thrush hosts (Grim, 2006) (this is also true for parasitic cowbird chicks in American robin nests: Croston & Hauber, 2015), but even partially misdirected parental care is costly to hosts as it may decrease the growth and survival of their own chicks in addition to energy spent on feeding foreign chicks (Samaš et al., 2014b; also see Croston & Hauber, 2015). Thrushes are thus selected to reject foreign eggs before cuckoos hatch and become competitors (or killers) of their own offspring. If thrushes cannot provide cuckoo chicks with sufficient parental care to assure the parasites' survival (Grim, 2006) and also face selective pressure to reject foreign eggs, cuckoos should not regularly parasitize thrush nests and, therefore, thrushes would not be likely to evolve specific cues for egg‐rejections to respond to cuckoo parasitism specifically (Samaš et al., 2014a).

Common cuckoos exhibit a plumage that mimics the distinctive barred bellies of sparrowhawks, sufficient to receive similar responses from species that are not hosts for the cuckoo but are prey for the sparrowhawk (Post & Götmark, 2006). This finding suggests that in species where such discrimination between cuckoo and sparrowhawk is possible, it may be an evolved response to cuckoo parasitism (Davies & Welbergen, 2008). Sparrowhawks are a regular predator of adult blackbirds, and playback of sparrowhawk calls can lead to lower egg rejection by blackbirds, supporting the hypothesis that cuckoo mimicry of these birds may aid cuckoos in parasitism of blackbirds through fear (Roncalli et al., 2019). In systems where such a coevolutionary relationship exists, hosts may still confuse nonthreatening birds resembling the cuckoo for the parasitism threat of a cuckoo based on a continuum of similarity in traits (Grim, 2005). Røskaft et al. (2002) found that blackbirds behaved aggressively towards a model cuckoo, though less so than suitable cuckoo hosts, and (categorized in the “large egg and nest” host type; pg. 622) at a similar level to unsuitable hosts. Aggression by blackbirds toward cuckoo dummies in this experiment was also found to be higher in areas of sympatry. Grim et al. (2011) find no significant difference in blackbirds' responses under sympatry or allopatry with cuckoos, as well as a roughly similar level of aggression towards both cuckoo and hooded crow *Corvus cornix* dummies. Further, blackbirds react aggressively to both cuckoo and visually similar (but nonthreatening) dummies such as of feral pigeons (*Columba livia*) (Grim & Honza, 2001). Moksnes et al. (1991) suggest that blackbird egg rejection behavior is not readily explained by current interspecific brood parasitism (since this occurs at a very low level) or by status as a formerly parasitized host having successfully evolved a rejection strategy (since other species in this category are able to recognize and reject both mimetic and nonmimetic eggs, and blackbirds are more likely to accept mimetic eggs). These suggestions support the possibility that blackbirds are suitable hosts for parasitism by other blackbirds and that their nest and egg rejection defenses have evolutionary origins in conspecific brood parasitism.

In turn, Ruiz‐Raya et al. (2016) found some rejection of mimetic eggs, and high or complete rejection of nonmimetic eggs painted to resemble those of cuckoos. The rejection of mimetic eggs was not increased by the presence of a blackbird dummy, and cuckoo dummies elicited significantly more aggression than did female blackbird dummies. Cuckoo dummies led to more inspection of eggs; all lines of evidence taken to support the evolution of rejection in response to interspecific brood parasitism. Soler (2014) argues that the low levels of intra/conspecific brood parasitism observed in blackbirds is not sufficient to be a driving selective force in egg rejection and that aggression towards cuckoo dummies is suggestive of a coevolutionary relationship (but see Samaš et al., 2014b). Finally, though both male and female blackbirds are capable of egg rejection, there may be sex dimorphism in their ability to recognize mimetic parasitic eggs. Accordingly, Ruiz‐Raya et al. (2019) found that females, but not males, were able to recognize and reject more mimetic eggs.

### 4.1 Implications for future research

Blackbirds are able to nest successfully in a range of habitats and appear to be among the generalist species relatively well‐suited to increasingly anthropogenic environments (e.g., Faivre et al., 2001; Hatchwell et al., 1996). They are common in their native range (Moskát et al., 2003), as well as successful invasives (Hale & V. Briskie, 2007; Kentish et al., 1995). Because they are intermediate egg rejectors, they represent a useful model for intraspecific variation in the rejection decision, and may have implications (e.g., Grim & Honza, 2001) for the egg recognition‐ and rejection‐related cognition of other thrushes (e.g., Turner & Hauber, 2021).

While males have been found to recognize and reject parasitic eggs (Ruiz‐Raya et al., 2019), it remains unclear what egg traits or experiences are used by males in egg ejection. Among many brood parasite hosts studied, females are typically the incubating sex (Lee et al., 2005) and have, as a result, been the focus of egg discrimination research. Nevertheless, males in several host clades have been observed ejecting the eggs of their corresponding brood parasite (Lee et al., 2005). Davies and Brooke (1988) observed male reed warbler (*Acrocephalus scirpaceus*) rejection of cuckoo egg models, though the presence of males at the nest was not as strong a predictor of rejection as the presence of females. Male rufous horneros (*Furnarius rufus*) (Tosi‐Germán et al., 2020) and Northern orioles (*Icterus galbula*) (Sealy & Neudorf, 1995) eject brown‐headed cowbird eggs, and male vinous‐throated parrotbills *Paradoxornis webbianus* reject common cuckoo eggs (Lee et al., 2005). Lee et al. (2005) suggest that since own egg color is likely to be consistent for a given female throughout lifetime (e.g., Hauber et al., 2019), while males may be exposed to different egg colors produced by different female mates, there is less selective pressure on males to discriminate based on color consistency.

Although there is no known cuckoo gens mimicking blackbird eggs in both color or size, it would be interesting to decouple these two traits in more experiments, as well as to more directly test features such as maculation (e.g., Hauber et al., 2014), intraspecific color variation (e.g., Hanley et al., 2017), or cloacal or uropygial chemical cues (e.g., Hauber, 2020; Soler et al., 2014; Turner et al., 2022). Multimodal recognition of cues has been tested relatively rarely in this species (e.g., Ruiz‐Raya et al., 2015; Soler et al., 2015), or to our knowledge, any of their congeners (e.g., Turner & Hauber, 2021). Turner et al. (2022) found that eggshell texture influenced the rejection of models by American robins, but note that it is, as in the case of egg size, not possible to isolate visual and tactile cues completely in these experiments. Multimodal information may be assembled by the receiver such that there is minor enhancement (the overall information drawn from the combined cues is greater than that of a cue in isolation, but less than the accumulated total), summation (direct addition), or multiplicative enhancement (where the total assembled information is greater than from each cue in isolation) (Partan, 2013). By isolating traits more precisely, future work could explore the relative influence of different sensory modalities in the blackbird's umwelt, as well as examining the structure of multimodal enhancement in egg recognition.

There have been tests of interpopulation variation based on proximity and sympatry to and with cuckoos (e.g., Moskát et al., 2003; Samaš et al., 2014a), but to our knowledge, there has been no attempt to record intergenerational similarities and potential heritability in egg rejection decision making. This may be prohibitively difficult with low nestling survival and/or low natal philopatry, but would be a worthwhile consideration in assessing the rate of change in this behavior at the individual lineage and at the population levels.

The hypothesis that the evolutionary ghost of a now‐extinct cuckoo gens evolved to parasitize turdids drives the genus' present resistance to parasitism remains feasible. As the phylogeny of thrushes becomes increasingly resolved (Nagy et al., 2019; Voelker et al., 2007), however, we may develop a clearer picture of how anti‐parasitism strategies are distributed in the clade and which species currently exhibit intraspecific/conspecific brood parasitism. Future work would benefit from mapping the cognitive/sensory traits and behavioral anti‐parasite strategies with the occurrence of intra/conspecific brood parasitism in the genus.

## CONFLICT OF INTEREST

The authors declare there is no conflict of interest.

## AUTHOR CONTRIBUTIONS

**Andrew G. Fulmer:** Data curation (lead); Formal analysis (lead); Investigation (lead). **Mark E. Hauber:** Conceptualization (lead); Funding acquisition (lead); Methodology (lead); Project administration (lead).

## Data Availability

Data sharing not applicable to this article as no datasets were generated or analyzed during the current study.
